# Malocclusion characteristics amongst individuals with autism spectrum disorder: a systematic review and meta-analysis

**DOI:** 10.1186/s12903-022-02366-0

**Published:** 2022-08-10

**Authors:** Thiago Peixoto da Motta, Janine Owens, Lucas Guimarães Abreu, Suélen Alves Teixeira Debossan, Fabiana Vargas-Ferreira, Mario Vianna Vettore

**Affiliations:** 1 Department of Social and Preventive Dentistry, School of Dentistry, Federal University of Minas Gerais, Av. Antônio Carlos, 6627 - Pampulha, Belo Horizonte, MG CEP 312270-901 Brazil; 2 NIHR Applied Research Collaborative, Greater Manchester (NIHR ARC GM), Faculty of Biology, Medicine and Health, Division of Nursing, Midwifery and Social Work, Jean McFarlane Building, University of Manchester, Oxford Road, Manchester, M13 9PL UK; 3 Department of Child and Adolescent Oral Health, School of Dentistry, Federal University of Minas Gerais, Av. Antônio Carlos, 6627 - Pampulha, Belo Horizonte, MG CEP 312270-901 Brazil; 4 Department of Health and Nursing Sciences, Faculty of Health and Sports Sciences, University of Agder, Campus Kristiansand, Universitetsveien 25, 4630 Kristiansand, Norway

**Keywords:** Autistic disorder, Malocclusion, Angle Class II, Malocclusion, Angle Class III, Open bite, Systematic review, Meta-analysis

## Abstract

**Background:**

To estimate the prevalence of malocclusion in individuals with autism spectrum disorders (ASD) and to assess the relationship between ASD and malocclusion.

**Methods:**

We searched electronic databases including PubMed, Scopus, Web of Science, Cochrane, Embase, SciELO LILACS, Proquest, OpenGrey and Google Scholar. There were no language or publication dates restrictions. Two researchers independently performed selection, data extraction and quality assessment. Quality assessment and risk of bias were evaluated through the Newcastle–Ottawa scale and ROBINS-E tool. Meta-analyses using random effect models were used to estimate pooled measures of prevalence of malocclusion characteristics in individuals with ASD and pooled odds ratio (OR) on the relationship between ASD and malocclusion characteristics. Subgroup meta-analyses were conducted according to children and adolescents, history of orthodontic treatment, and occurrence of other syndromes and medical conditions.

**Results:**

Searching identified 5549 papers with 238 were selected for full assessment. Eighteen cross-sectional studies were included according to inclusion criteria. Of them, eleven studies were considered of moderate quality. A judgement of critical risk of bias occurred for thirteen studies. The most prevalent malocclusion characteristics in individuals with ASD were crowding (33%; 95% CI 22 to 44%) and increased maxillary overjet (39%; 95% CI 23 to 54%). Individuals with ASD had higher odds of Angle’s Class II (OR 1.92; 95% CI 1.36 to 2.72), Angle’s Class III (OR 2.33; 95% CI 1.29 to 4.23), open bite (OR 1.96; 95% CI 1.21 to 3.16), and increased maxillary overjet (OR 1.53; 95% CI 1.06 to 2.21) than individuals without ASD.

**Conclusions:**

Angle’s Class II, Angle’s Class III, anterior open bite and increased maxillary overjet were more prevalent in individuals with ASD than those without ASD. Further high-quality studies are needed.

## Background

Autism spectrum disorder (ASD) is a lifelong and complex developmental condition linked to the atypical neurodevelopment usually diagnosed between the ages of one to six years depending on access to healthcare services [1]. It is estimated that 1 in 270 people have ASD, with abilities and needs varying between individuals from those living independently with minimum support to those requiring lifelong care [2]. Environmental and genetic factors have previously been linked to the occurrence of ASD, although the aetiologic mechanisms remain unknown [3]. Individuals with ASD may experience persistent challenges in social interaction and communication [4]. Intellectual disability is often a coexisting condition in approximately 50% of individuals with ASD and frustration with communication challenges, coupled with an unsupportive environment may often lead to behavioural outbursts [5, 6]. The dimensions of social interaction and communication as well as restrictive and repetitive behaviour are part of the assessment procedure for ASD in The Diagnostic and Statistical Manual of Mental Disorders (DSM-5), although diagnosis is not always straightforward [7, 8].

Recent evidence suggests that a diagnosis of ASD may be accompanied by the occurrence of dental problems and health impairing behaviours, such as poor oral hygiene, which predisposes individuals with ASD to gingivitis and poorer periodontal health [9, 10]. Individuals with ASD experience increased rates of immunological and gastrointestinal problems, sleeping disorders, mental health problems, convulsion, obesity, hypertension, and diabetes [11]. A previous systematic review including ten primary studies indicates a lack of consensus whether the incidence of dental caries is higher among people with ASD [9]. Furthermore, children with ASD present with greater prevalence of halitosis, oral lesions, and dental pain and many individuals with ASD have at least one dental problem creating negative impacts on their quality of life [12]. Pharmacological interventions for people with ASD and coexisting conditions often control behaviour [13]. Side effects of some of the drugs are gingival bleeding, gingival overgrowth, hyperplasia, aphthous ulcers, delayed healing, and xerostomia [14]. The associated challenges may lead to poorer oral health, often compounded by the lack of effective health promotion for individuals with ASD and their carers compared to individuals without ASD, resulting in an increased demand and use of health services [15, 16].

For individuals without ASD, malocclusion is a craniofacial developmental disorder affecting teeth, bones, and facial muscles. The multifactorial aetiology of malocclusion includes genetic and environmental factors as well as persistent harmful oral habits [17, 18]. A previous systematic review revealed the global prevalence of Angle’s Class I, Class II and Class III as 74.7%, 19.6% and 5.9%, respectively. In addition, an increased maxillary overjet and deep overbite were estimated as 20.1% and 22.0%. The prevalence of open bite and posterior cross bite were 4.9% and 9.4% [18]. The negative impact of malocclusion on quality of life has been extensively reported in children. Children without ASD and diagnosed with malocclusion perceive more functional problems, including speaking, chewing, and sleeping, as well as impacts affecting social interaction, self-esteem, and oral health satisfaction [19].

ASD is a diverse condition and there appear to be morphological facial differences arising from genetic mechanisms for some individuals [20]. For example, fragile X syndrome is associated with ASD, with studies indicating a higher occurrence of malocclusion among individuals with the syndrome [21]. However, testing for fragile X remains a subject for debate because there is no available treatment and it may be unknown whether an individual with ASD also has fragile X [22]. Another overlapping syndrome with ASD is Rett syndrome, often misdiagnosed as ASD and can occur as a syndrome without ASD [23]. A systematic review on oral health and Rett syndrome suggested a higher prevalence of anterior open-bite and mouth breathing in affected individuals, but the study did not identify whether there was an interplay with ASD [24]. Another syndrome associated with ASD is Phelan-McDermid with a high frequency of malocclusion [25]. The genetic landscape of ASD and its association with other syndromes appears inconclusive and complex.

Harmful oral habits, including para-functional habits, are more common in individuals with ASD than those without [26]. Compared to controls, individuals with ASD reported greater prevalence of bruxism, mouth breathing, biting objects, lips or tongue, nail biting and finger sucking [12, 26]. The influence of harmful oral habits on malocclusion and the greater prevalence of para-functional oral habits in individuals with ASD raises the question as to whether ASD predisposes distinct types of malocclusions. Therefore, the aims of this study were to systematically review the existing literature on the prevalence of the different malocclusion characteristics in individuals with ASD and to examine the association between ASD and malocclusion.

## Methods

### Protocol registration

The protocol for the present systematic review was registered on the National Institute of Health Research Database (registration number CRD42019151794; http://www.crd.york.ac.uk/PROSPERO).

### Eligibility criteria

The studies included in this systematic review met the following selection criteria. (1) Participants: Individuals of any age group who had or not had undergone previous orthodontic treatment. (2) Exposure: Individuals with ASD diagnosis. (3) Comparator: Studies had to report at least one malocclusion characteristic of individuals diagnosed with ASD. They could include one or more comparison groups such as individuals without ASD or individuals with other syndromes or intellectual disabilities. (4) Outcome measures: Malocclusion characteristics on clinical examination was the main outcome. The condition must have been assessed through clinical visual inspection using malocclusion indices such as the Dental Aesthetic Index (DAI), clinical classifications, such as Angle’s Class, or through the presence of horizontal or vertical malocclusions. (5) Study design: Prospective or retrospective cohort studies, case–control and cross-sectional studies were retrieved for inclusion. Ineligible papers included interventional studies and previous review papers.

### Literature search strategy and selection of papers

Databases searched included PubMed, Scopus, Web of Science, Cochrane, Embase, SciELO and LILACS, up to November 2021. Grey literature was examined through Proquest, OpenGrey and Google Scholar. There were no language restrictions. The electronic searches were carried using a combination of search terms linked through Boolean operators (Table 1). Manual searching took place of the reference lists of included articles and those from previously identified systematic and narrative reviews.

**Table 1:** Study search strategy

Search groups	(1)	(2)
Key-words	(a) Malocclusion Malocclusion, angle class Malocclusion, angle class II malocclusion, angle class III (b) Orthodontics Orthodontic, corrective Index of orthodontic treatment needs Dental aesthetic index Stomatognathic System Abnormalities Stomatognathic diseases Tooth Abnormalities Dental Care for disabled Dental care, disability	Handicapped Mentally handicapped Learning disability* Intellectual disability* (c) Asperger’s Neurodiversity Child development disorders, pervasive (d) Autism Autism spectrum disorders Autistic disorder Neurodevelopmental disorders
Database	Search strategy	
PubMed	(1 AND 2)	
Scopus	(1 AND 2)	
Web of Science	(1 AND 2)	
Cochrane	(1 AND 2)	
Embase	(1 AND 2)	
Scielo	(1 AND 2)	
Lilacs	(1 AND 2)	
Proquest	(a OR b AND c OR d)	
OpenGrey	(a OR b AND c OR d)	
Google Scholar*	(a OR b AND c OR d)	

*On Google Scholar database search, only the first hundred hits were considered

Total: 5549

Titles and abstracts of all retrieved papers were independently screened and selected for inclusion by two authors (T.P.M. and S.A.T.). A third author (M.V.V.) who did not participate in the original screening and selection of papers was involved in the discussion to resolve any disagreements.

### Data extraction

Relevant data of included papers were independently extracted in duplicate by two authors (T.P.M. and M.V.V.). The following information was recorded: (1) author and year of publication; (2) study design; (3) country; (4) study setting; (5) participants: sample size, gender rate, participant’s age; (6) malocclusion measures, including examiners’ background, clinical calibration, and examination conditions; (7) eligibility criteria; and (8) comparison group.

### Quality assessment

The quality assessment was carried out independently by two authors (T.P.M. and M.V.V.) using the Newcastle–Ottawa scale (NOS) [27]. Any disagreements were resolved by consensus. The NOS evaluates the methodological quality of individual studies following a star system based on 8 domains grouped into 3 main domains: patient selection, comparability of study groups, and outcome assessment. Cohort and case–control studies may receive up to 9 stars and cross-sectional studies may receive up to 10 stars. Studies were categorized as high-quality, moderate quality and low quality if they reached 7–9 (cohort and case–control studies) or 7–10 (cross-sectional studies) stars, 4–6 stars and 0–3 stars, respectively.

### Risk of bias of individual studies

The risk of bias of individual studies was carried out independently by two authors (T.P.M. and S.T.D.) using the Risk of Bias in Nonrandomized Studies of Exposures (ROBINS-E) tool [28]. Any disagreements were resolved by consensus with input from a third reviewer (M.V.V.). The application of the Risk of bias (RoB) instrument followed the three steps. In the first step, reviewers revised the review question and specific aspects of sources of bias, such as confounders, and exposure and outcome measurements. The second step involved the description of a hypothetical ideal study and specific confounders. Finally, each study was compared to the ideal study considering the RoB criteria across the seven items: (1) bias due to confounding, (2) bias in selection of participants into the study, (3) bias in classification of exposures, (4) bias due to departures from intended exposures, (5) bias due to missing data, (6) bias in measurement of outcomes, (7) bias in selection of the reported result. Initially, the examiners answer the ROBINS-E questions using the options “yes,” “Probably yes,” “Probably no,” or “No.” Then, each RoB item was assessed as ‘low,’ ‘moderate,’ ‘serious,’ or ‘critical,’ to judge RoB at study-level and at item-level.

### Quantitative synthesis and statistical analysis

Meta-analyses were conducted to obtain summary measures (prevalence) and pooled effect sizes (odds ratios) and 95% confidence interval (CI) using random effects models to account for the heterogeneity between primary estimates. Both pooled prevalence measures and pooled odds ratios were estimated using the inverse variance method. Producing forest plots related to the different malocclusion classifications (e.g., Angle’s classification, Dental Aesthetic Index (DAI)) and malocclusion characteristics. Meta-analyses were conducted for all studies that provided data. Sub-group analyses were conducted for (i) studies including only children and adolescents, (ii) studies excluding individuals with history of orthodontic treatment, and (iii) studies that excluding or providing information about other syndromes and medical conditions.

To obtain pooled prevalence, the original estimates were submitted to logit transformations to account for the distribution asymmetry. Then, the transformed estimates were weighted by the logit. The pooled prevalence estimates were generated thereafter. The following definitions of malocclusion characteristics were used in the meta-analyses of prevalence and in those comparing malocclusion characteristics between individuals with ASD and without ASD: increased maxillary overjet ≥ 3 mm, anterior cross-bite ≥ 0 mm, and open bite ≥ 0 mm [29].

Studies comparing malocclusion measures between individuals with ASD and those without any deficiency were included to obtain pooled effect sizes. Studies reporting an odds ratio and 95%CI were reported or could be obtained through numerical transformation using continuous measures (e.g., mean differences, correlations) were included [30]. I^2^ statistics assessed the proportion of the variance due to statistical heterogeneity among studies comparing malocclusion measures between individuals with and without ASD [31]. Meta-analyses reporting I^2^ as equal or less than 50% acknowledged heterogeneity [32]. Assessing heterogeneity in the studies reporting the prevalence of malocclusion among individuals with ASD occurred through prediction intervals (PI) [33]. The decision not to conduct a publication bias assessment resulted from power issues, because only one meta-analysis included more than 10 studies [34]. All statistical analyses were performed using Stata software (version 16.0) using the commands ‘metaprop’ and ‘metan’ to obtain pooled prevalence estimates and pooled effect sizes.

## Results

### Study selection

**Fig. 1:**
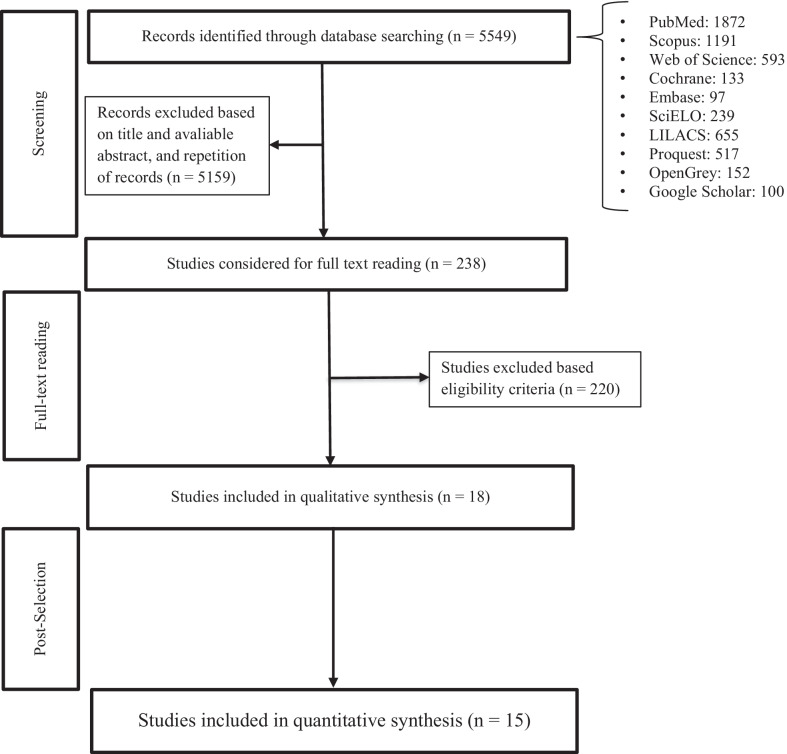
Flow chart of studies identification and selection

A PRISMA flow chart reports the number of outputs retrieved, screened, and selected (Fig. 1). The initial electronic search yielded 5549 articles after removing duplicates. The search of references did not retrieve any further relevant publications. After the initial screening of titles and abstracts 238 articles were selected for full assessment. After the full-text analysis, 18 articles assessing 2194 individuals with ASD and 10,846 without ASD were included in the systematic review [35–51]. The Kappa coefficient regarding the agreement between authors involved in selection of papers was 0.70.

### Characteristics of included studies

The characteristics of the included studies are summarized in Table 2. Eighteen cross-sectional studies were identified. Of these, two studies, originally classified as case–control studies [40, 44] selected participants with a diagnosis of ASD (exposure of interest in this study) and reclassified as cross-sectional studies. Three studies included individuals solely with ASD [41, 50, 51]. Included studies from 13 countries selected participants from rehabilitation centres, healthcare services, schools for disabled children, university dental hospitals and mainstream schools. Sample size for the studies ranged from 54 to 844. Of the 18 studies, 13 assessed children and adolescents up to 18 years of age. Only five studies excluded individuals with a history of orthodontic treatment [38, 43, 45, 46, 52]. The occurrence of other syndromes and medical conditions were considered in eight studies [35, 36, 39, 42, 43, 45, 46, 52]. Different malocclusion measures and malocclusion indices used included Angle’s classification, DAI, crowding, posterior crossbite, increased maxillary overjet, anterior crossbite, open bite and deep bite.

**Table 2:** Characteristics of selected studies (N = 18)

References	Study design	Country	Setting	Participants	ASD diagnosis	Malocclusion measures	Eligibility criteria*	Group of comparison
Vittek et al. [35]	Cross-sectional	United States	Healthcare services	N total = 458; N ASD = 26 boys (63.8%), girls (36.2%) Age group: 6–87 years-old	Medical records	Angle's classification, crowding, crossbite, open bite, overbite and overjet Examiner: N.I Clinical calibration: N.I Examination conditions: dental chair using mouth mirroe and probe	*Exclusion criteria* Edentulous patients and those without molar relationship	Yes Children without intellectual and mental disabilities (N = 8841), organic brain (N = 238), seizure disorder (N = 90), cerebral palsy (N = 47), down (N = 57)
Manzano et al. [36]	Cross-sectional	Venezuela	Special education institutes	N total = 133; N ASD = 23 boys (55.6%), girls (44.4%) Age group: 3–4 years-old	Medical diagnosis	Angle's classification (só relata no resultado) Examiner: N.I Clinical calibration: N.I Local of examination: N.I	*Inclusion criteria* Age between 3 and 4 years	Yes Down syndrome (N = 65), deaf and speech impaired (N = 26), sight impaired (N = 7), cerebral palsy (N = 12)
DeMattei et al. [37]	Cross-sectional	United States	Three schools for disabled children	N total = 55; N ASD = 39 boys (72.7%) girls (27.3%) Age group: 2.6–21.0 years-old	Medical records and school files	Angle´s classification, crowding, crossbite Examiner: dental hygienists Clinical calibration: N.I Examination conditions: portable dental chairs	None	Yes Children with other developmental disorders (N = 16)
Luppanapornlarp et al. [38]	Cross-sectional	Thailand	Division of Dentistry of University	N total = 80; N ASD = 32 boys (78.1%), girls (21.8%) Age group: 8–12 years-old Mean age = 9.8 ± 1.1	Not reported	Dental Aesthetic Index Examiner: N.I Clinical calibration: Intrarater Correlation Coefficient = 0.98 Examination conditions: N.I	*Inclusion criteria* Age between 8 and 12 years, history of orthodontic treatment *Exclusion criteria* Inability to cooperate in the oral examination	Yes Children without ASD (N = 48)
Soni et al. [39]	Cross-sectional	India	Special schools	N total = 78, N ASD = 10 boys (66.7%) and girls (33.3) Age group:12–15 years	Not reported	IOTN Examiner: N.I Clinical calibration: Kappa > 0.82 Examination conditions: use of natural daylight	*Exclusion criteria* Age between 12 and 15 years, Inability to cooperate in the oral examination	Yes Down syndrome (N = 4), hearing impaired (N = 11), learning disability (N = 2), mental retardation (N = 43), orthopedic disability (N = 2), spastic paraplegia (N = 1) and visually impaired (N = 5)
Orellana et al. [40]	Cross-sectional	Spain	Two day-centres for people with autism	N total = 60; N ASD = 30 boys (90.0%), girls (10.0%) Age group: 20–41 years-old Mean age = 27.8 ± 5.8	Not reported	Dental crowding, open bite Examiner: dentists Clinical calibration: Kappa > 0.81 Examination conditions: portable dental chair and lamp	None	Yes Individuals without ASD (N = 30)
Rekha et al. [41]	Cross-sectional	India	Twelve special education schools, three autistic child centres and three therapy centres	N ASD = 483 boys (75.1%), girls (24.9%) Age group: 4–16 years-old	School files	Proclination, crowding, anterior open bite, rotation Examiner: pediatric dentistry Clinical calibration: N.I Local of examination: mouth mirror in broad daylight	None	No
Muppa et al. [42]	Cross-sectional	India	Eleven special schools	N total = 844; N ASD = 40 boys (80.0%), girls (20.0%) Age group: 6–30 years-old	Not reported	Class I, Class II, Class III, anterior crowding, anterior spacing, deep bite, open bite, and anterior cross bite Examiner: N.I Clinical calibration: N.I Examination conditions: under natural light, with the child in the knee to knee position	None	Yes Mild Intellectual Disability (MID) (N = 308), moderate ID (N = 201), severe ID (N = 83, Hearing and speech (N = 172), cerebral palsy (N = 40)
Vellappally et al. [43]	Cross-sectional	India	Fourteen special schools for disabled	N total = 243; N ASD = 14 boys (60.1%), girls (39.9%) Age group: 12–18 years-old Mean age 14.1 ± 2.0	Not reported	Dental Aesthetic Index Examiner: N.I Clinical calibration: reproducibility = 90% Examination conditions: N.I	*Inclusion criteria* Ages ≥ 12 and < 19 years, intelligence Quotient (IQ) ≤ 85 *Exclusion criteria* Inability to cooperate in the oral examination, history of orthodontic treatment	Yes Intellectual disability (ID) alone (N = 108), ID and cerebral palsy (N = 55), Down’s syndrome (N = 36), ID and a learning disability (N = 18) and ID and a speech-hearing impairment (N = 12)
Du et al. [44]	Cross-sectional	China	Nineteen special child care centres	N total = 514; N ASD = 257 boys (84.4%), girls (15.6%) Age group: 2.7–6.4 years-old Mean age = 4.9 ± 0.8	Not reported	Deep overbite, anterior open bite, increased overjet, anterior crossbite and posterior crossbite Examiner: N.I Clinical calibration: Kappa > 0.70 Examination conditions: chair using an intra-oral mirror with a LED light source	None	Yes Children without ASD (N = 257)
Alkhadra [45]	Cross-sectional	Kingdom of Saudi Arabia	Five rehabilitation centres for disabled children	N total = 72; N ASD = 55 boys (72.7%) girls (27.3%) Age group: 6–14 years-old	Medical records	Angle’s classification, overjet, overbite, incisor open bite, cross bite in the right and left side on both anterior and posterior Examiner: N.I Clinical calibration: N.I Examination conditions: portable chair under natural light using a disposable mouth mirror and tongue blade	*Exclusion criteria* History of ongoing medical treatment, extraction, and orthodontic treatment	Yes Children with Down syndrome (N = 100)
Fontaine-Sylvestre et al. [46]	Cross-sectional	Canada	Division of dentistry, children's hospital	N total = 200; N ASD = 99 boys (78.8%), girls (21.2%) Age group: 5–18 years-old Mean age = 11.0 ± 3.7	Medical diagnosis	Angle's classification, midline deviation, crossbite, open bite, overbite, crowding, Examiner: N.I Clinical calibration: N.I Examination conditions: dental chair	*Inclusion criteria* Age between 5 to 18 years Mixed or permanent dentition *Exclusion criteria* Another disorder or syndrome than ASD, history of orthodontic treatment, Incomplete files with respect to the child's diagnosis of ASD	Yes Children without ASD (N = 101)
Alkhabuli et al.[47]	Cross-sectional	United Arab Emirates	Rehabilitation centres for disabled children	N total = 54; N ASD = 9 boys (70.4%) girls (29.6%) Age group: 3–17 years-old	Medical records	Angle’s classification, crowding; spacing, anterior open bite, IOTN Examiner: N.I Clinical calibration: Kappa = 0.83 Examination conditions: adjustable chair using torchlight, dental explorer, and mouth mirror	*Inclusion criteria* Age ≤ 17 years	No
Kuter and Guler [48]	Cross-sectional	Turkey	Regular schools	N total = 407; N ASD = 285 boys (80%), girls (20%) Age group: 5–16 years-old	Not reported	crowding, open bite, deep-palate Examiner: N.I Clinical calibration: N.I Examination conditions: chair using dental mirror and explorer	None	Yes Children without ASD (N = 122)
Leiva-Garcia et al. [49]	Cross-sectional	Spain	Rehabilitation centre for disabled children	N total = 146; N ASD = 55 boys (74%), girls (26%) Age group: 6–18 years-old Mean age = 10.7 ± 3.0	Medical diagnosis	Angle's classification, open bite, crossbite Examiner: one pediatric dentist Clinical calibration: Kappa = 0.80 Examination conditions: chair under natural light and using a sterile exploration kit comprising an oral probe and mirror, and cotton tips	*Exclusion criteria* Special diets, food allergies or medications capable of modifying dietary intake and oral health	Yes Children with typical development (N = 91)
Orellana et al. [50]	Cross-sectional	Chile	Institutions for people with ASD	N ASD = 123 boys (82.9%), girls (17.1%) Age group: 4–23 years-old Mean age = 9.4 ± 4.3	Not reported	Deep/ogival palate, anterior open bite, anterior and posterior crossbite Examiner: dentists Clinical calibration: Kappa > 0.81 Examination conditions: portable dental chair and lamp	*Inclusion criteria* Understanding very simple instructions	No
Mangione et al. [51]	Cross-sectional	France	special dental care department Division of Dentistry of University	N ASD = 118 boys (75.4%), girls (24.6%) Age group: 4–53 years-old Mean age = 23.3	Medical records	mild to severe dental and/or alveolar malocclusions Examiner: N.I Clinical calibration: N.I Examination conditions: dental chairs	None	No
Bagattoni et al. [52]	Cross-sectional	Italy	Special Needs Dentistry Unit of University	N total = 128, N ASD = 64 boys (66%), girls (34%) Age group: 9.0 ± 2.9 years	Medical diagnosis	Angle’s classification, posterior crossbite, anterior open bite and deep bite Examiner: dentists Clinical calibration: N.I Examination conditions: dental chair using a dental mirror and a WHO periodontal probe	*Exclusion criteria*: Medical condition associated with oral diseases, inability to cooperate in the oral examination, dental prophylaxis in the previous 6 months, history of orthodontic treatment	Children without ASD (N = 64)

*Diagnostic methods of ASD and non-ASDs were not considered as eligibility criteria

N.I.: not informed

### Quality assessment

The modified Newcastle–Ottawa scale for cross-sectional studies was used to score the methodologic quality (Table 3). Five studies achieved a maximum of 3 stars or less and were assigned as having low quality [39, 40, 42, 44, 50]. Eleven studies were considered as having moderate quality [35–37, 41, 43, 45, 47–49, 51, 52], and two studies were assessed as high quality [38, 46]. Fifteen studies achieved 2 or less stars for selection of the study groups. Only one study selected a representative sample. Two studies reached 2 stars for comparability and all studies achieved two or more stars for outcome.

**Table 3:** Quality assessment according to Newcastle–Ottawa of the included studies (n = 18)

References	Selection			Comparability		Outcome		Stars
	Representativeness of the sample	Sample size	Non-respondents	Ascertainment of the exposure	Control for confounders	Assessment of the outcome	Statistical test	
Vitek et al. [35]	b	b	c	a**	c	b**	b	4*
Manzano et al. [36]	c	a*	c	a**	c	b**	c	5*
DeMattei et al. [37]	c	b	c	a**	c	b**	c	4*
Luppanapornlarp et al. [38]	c	b	c	a**	ab**	b**	a*	7*
Soni et al. [39]	c	b	c	c	c	a**	c	2*
Orellana et al. [40]	c	b	c	c	b*	b**	b	3*
Rekha et al. [41]	c	b	c	a**	c	b**	c	4*
Muppa et al. [42]	c	b	c	c	c	b**	c	2*
Vellappally et al. [43]	c	b	c	a**	c	a**	c	4*
Du et al. [44]	c	b	c	c	b*	b**	b	3*
Alkhadra [45]	c	b	c	a**	c	b**	c	4*
Fontaine-Sylvestre et al. [46]	c	b	c	a**	ab**	b**	a*	7*
Alkhabuli et al. [47]	c	b	c	a**	c	b**	c	4*
Kuter and Guler [48]	b*	b	c	c	b*	b**	b	4*
Leiva-Garcia et al. [49]	c	b	c	a**	b*	b**	b	5*
Orellana et al. [50]	c	b	c	c	c	a**	c	2*
Mangione et al. [51]	c	b	c	a**	c	b**	c	4*
Bagattoni et al. [52]	c	b	c	a**	c	b**	c	4*

### Risk of bias of individual studies

The ROBINS-E tool was used to assess RoB (Table 4). Nine were judged as critical risk of bias and four at serious risk. All studies were at low risk of selection bias. Nine studies were at critical risk of bias due to the measurement of exposure and nine at moderate risk. The departure from exposure domain was not relevant for all studies. One study was at moderate risk of bias due to missing data, 12 at serious risk, and five at critical risk. Thirteen studies were at critical risk of bias due to measurement of outcomes and five studies at serious risk. Twelve studies were at moderate risk of bias due to the reported results, and six studies were at critical risk. Of the 18 studies, none was judged as of low risk of bias, one [52] was assessed as having a moderate risk of bias, four studies [35, 38, 47, 51] were assessed as having a serious risk of bias, and 13 at critical risk of bias.

**Table 4:** ROBINS-E risk of bias assessment

References	Confounding	Selection	Measurement of exposure	Departure from exposure	Missing data	Measurement of outcomes	Reported Results	Study-level RoB judgment
Vitek et al. [35]	C	L	M	NR	S	C	M	S
Manzano et al. [36]	C	L	C	NR	C	C	M	C
DeMattei et al. [37]	C	L	M	NR	C	C	M	C
Luppanapornlarp et al. [38]	S	L	C	NR	S	S	M	S
Soni et al. [39]	C	L	C	NR	S	S	C	C
Orellana et al. [40]	C	L	C	NR	S	C	M	C
Rekha et al. [41]	NR	L	C	NR	C	C	M	C
Muppa et al. [42]	C	L	C	NR	S	C	C	C
Du et al. [43]	C	L	M	NR	C	S	M	C
Vellappally et al. [44]	S	L	C	NR	S	S	M	C
Alkhadra [45]	S	L	M	NR	S	C	C	C
Fontaine-Sylvestre et al. [46]	S	L	M	NR	S	C	C	C
Alkhabuli et al. [47]	NR	L	M	NR	M	C	M	S
Kuter & Guler [48]	C	L	C	NR	C	C	C	C
Leiva-Garcia et al. [49]	C	L	M	NR	S	C	C	C
Orellana et al. [50]	NR	L	C	NR	S	C	M	C
Mangione et al. [51]	NR	L	M	NR	S	C	M	S
Bagattoni et al. [52]	M	L	M	NR	S	S	M	M

*L* low, *M* moderate, *S* Serious, *C* Critical, *NR* not relevant

### Prevalence of malocclusion in individuals with ASD

**Fig. 2:**
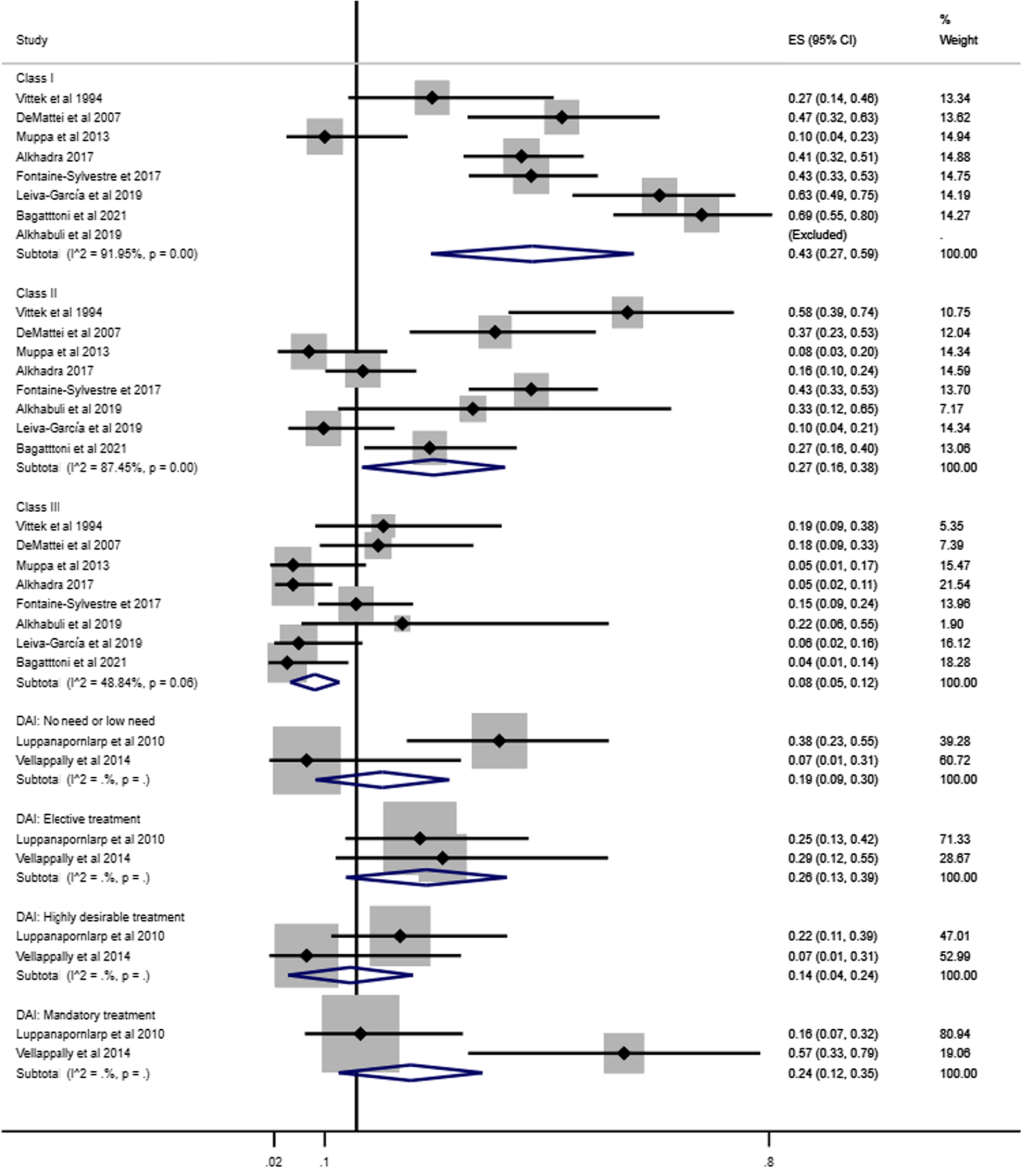
Forest-plot for prevalence of malocclusion according to Angle´s Class and DAI among individuals with ASD

**Fig. 3:**
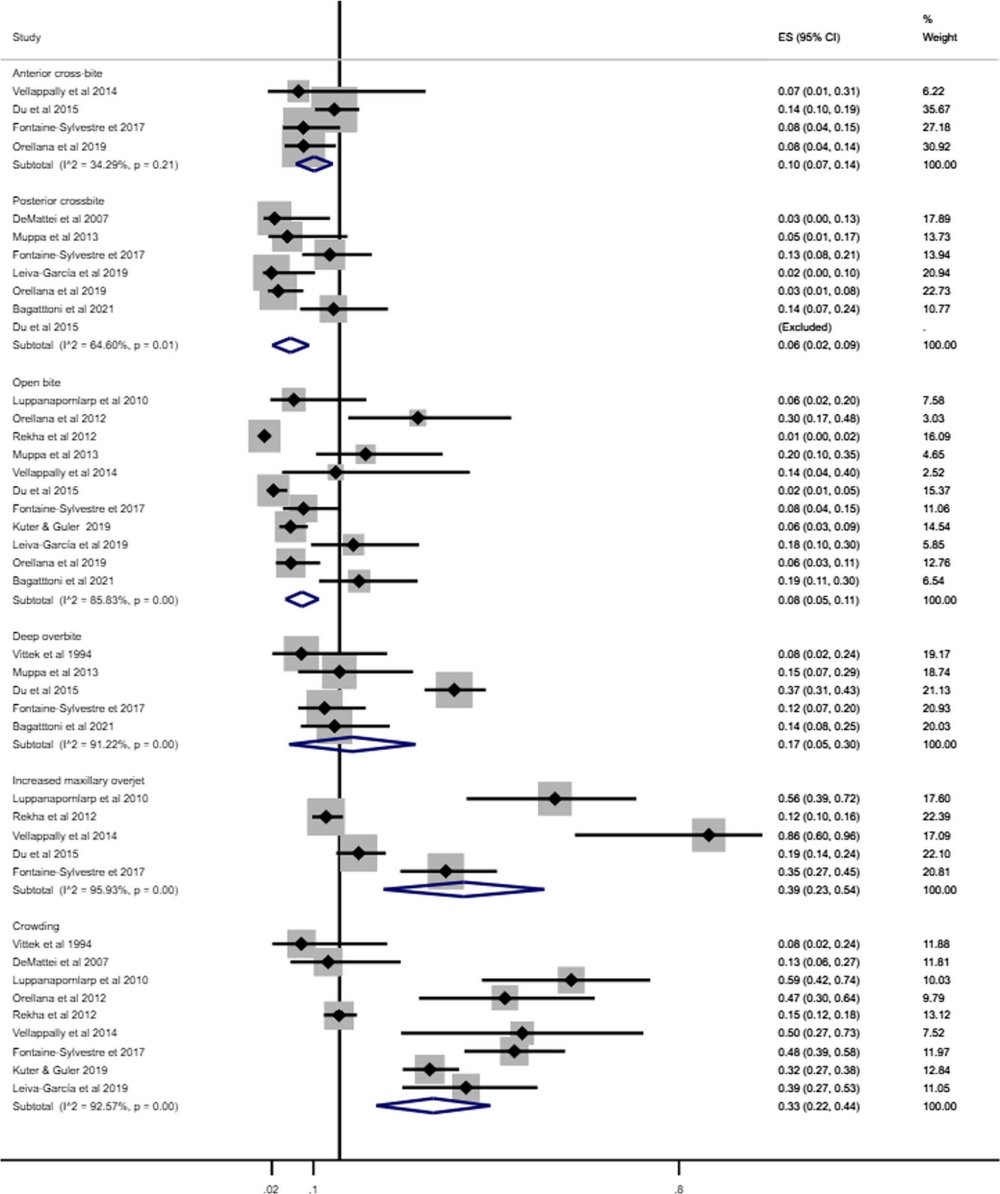
Forest-plot for prevalence of malocclusion characteristics among individuals with ASD

The forest plot combining the prevalence of malocclusion classifications (Angle’s Class and DAI) and characteristics of malocclusion in individuals with ASD derived from 15 studies involving 1458 individuals are shown in Figs. 2 and 3 [35, 37, 38, 40–50, 52]. The pooled prevalence of Angle’s Class I, Class II and Class III in individuals with ASD were 43% (95% CI 27%–59%), 27% (95% CI 16%–38%) and 8% (95% CI 5%–12%), respectively. The pooled measures of highly desirable treatment and mandatory treatment according to DAI were 14% (95% CI 4%–24%) and 24% (95% CI 12%–35%), respectively (Fig. 2).

Increased maxillary overjet (39%, 95% CI 23%–54%) and crowding (33%, 95% CI 23%–44%) were the most prevalent malocclusion characteristics in individuals with ASD. The least common malocclusion conditions were posterior crossbite (6%, 95% CI 2%–9%) and open bite (8%, 95% CI 6%–11%) (Fig. 3).

**Fig. 4:**
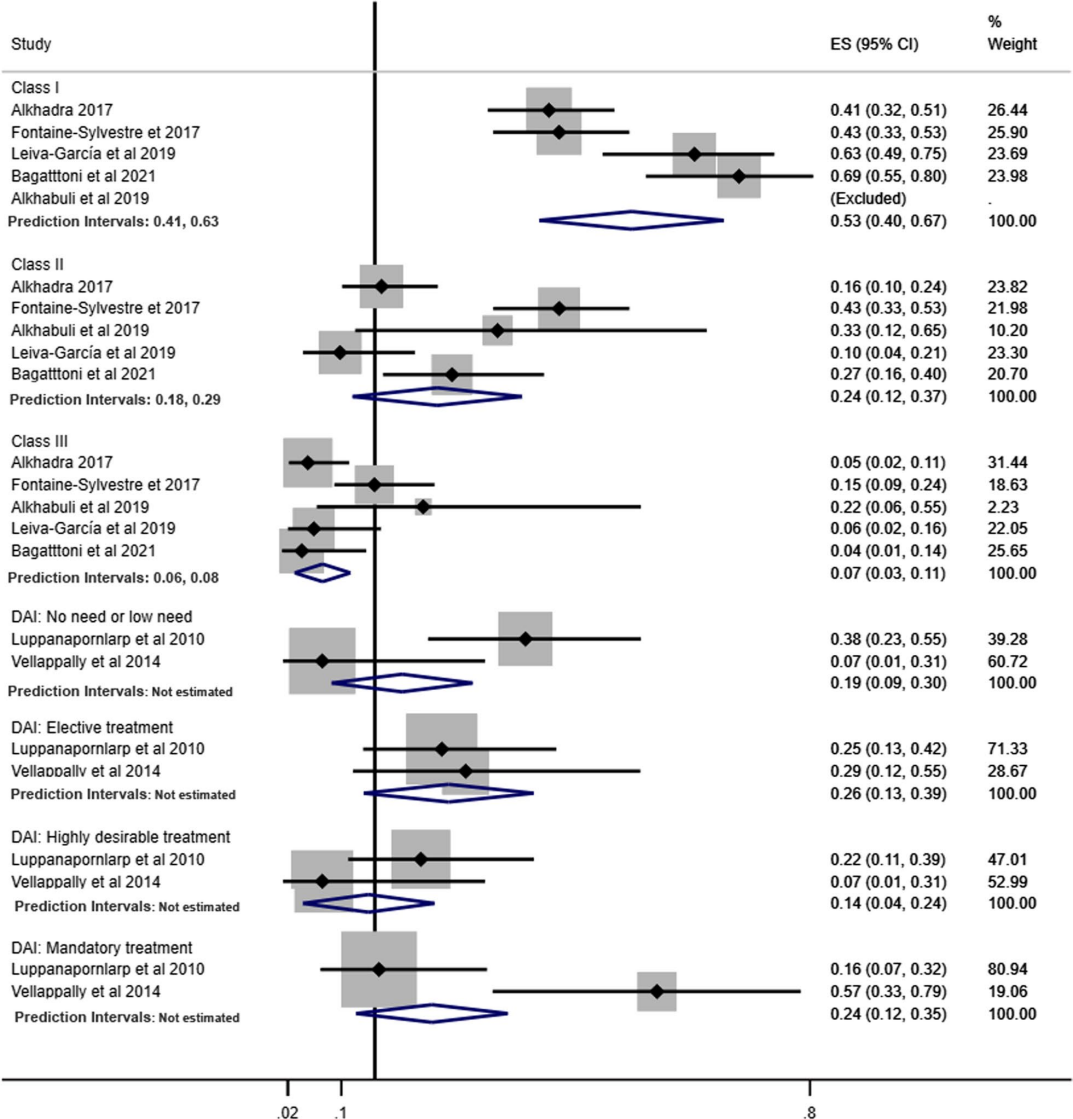
Prevalence of Angle’s Class and DAI in children and adolescents with ASD

**Fig. 5:**
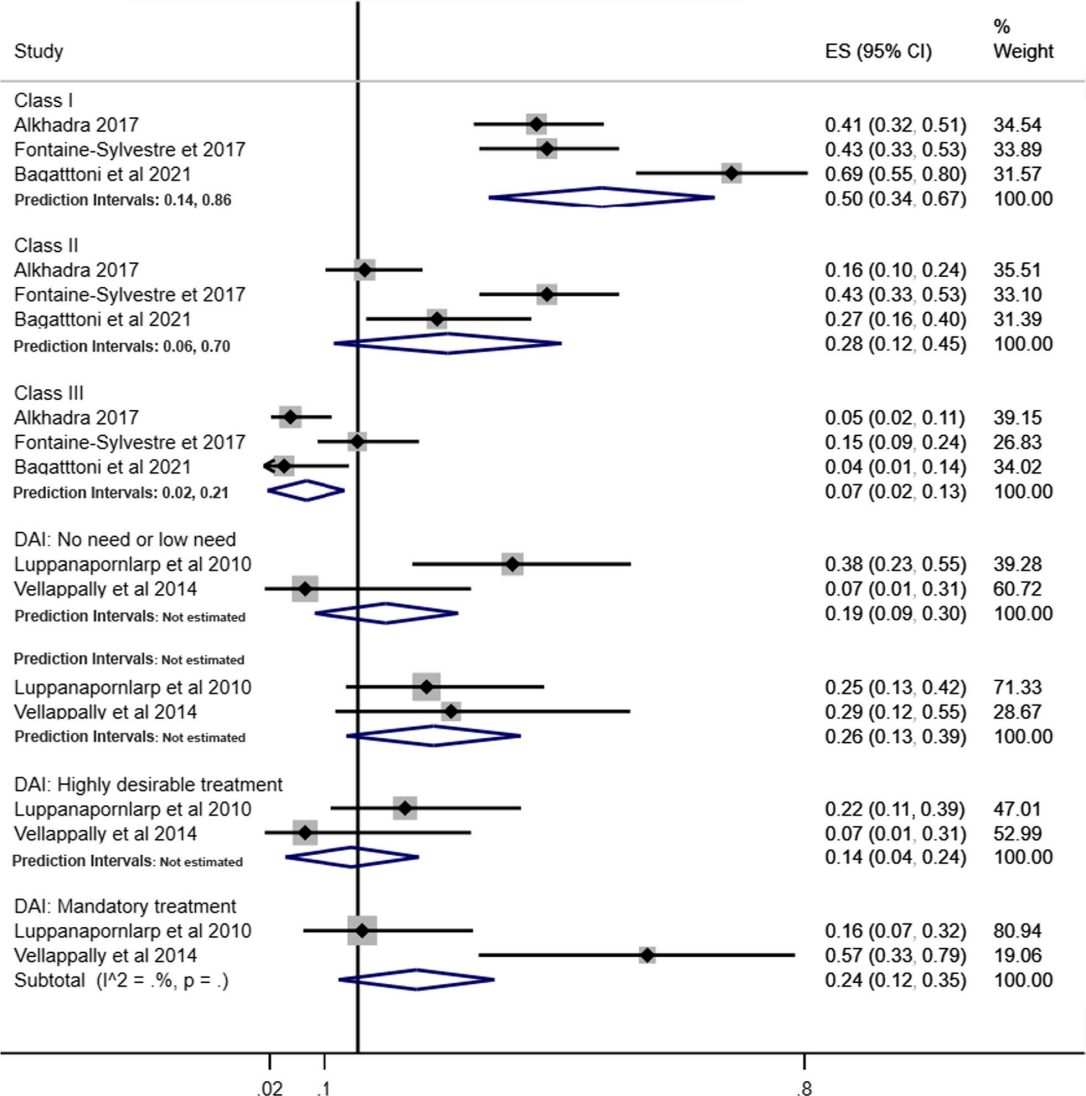
Prevalence of Angle’s Class and DAI in individuals with ASD without history of orthodontic treatment

**Fig. 6:**
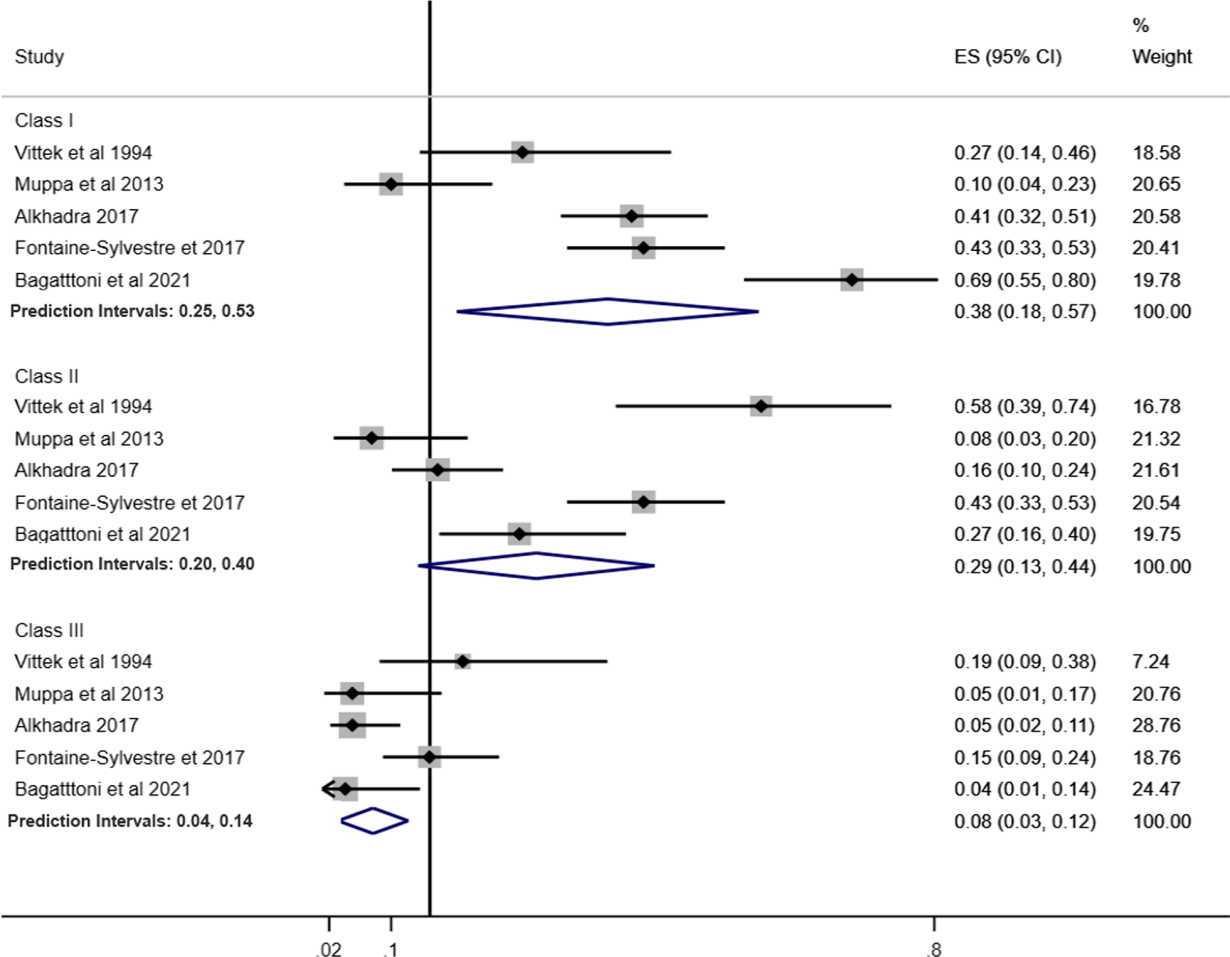
Prevalence of Angle’s Class and DAI in individuals with ASD involving studies that excluded or provided information about other syndromes and medical conditions

Pooled prevalence of Angle’s Class was estimated according to children and adolescents (Fig. 4), history of orthodontic treatment (Fig. 5) and studies that excluded or provided information about other syndromes and medical conditions (Fig. 6). The pooled estimates of DAI categories were obtained from two studies including only children and adolescents and participants without previous orthodontic treatment.

**Fig. 7:**
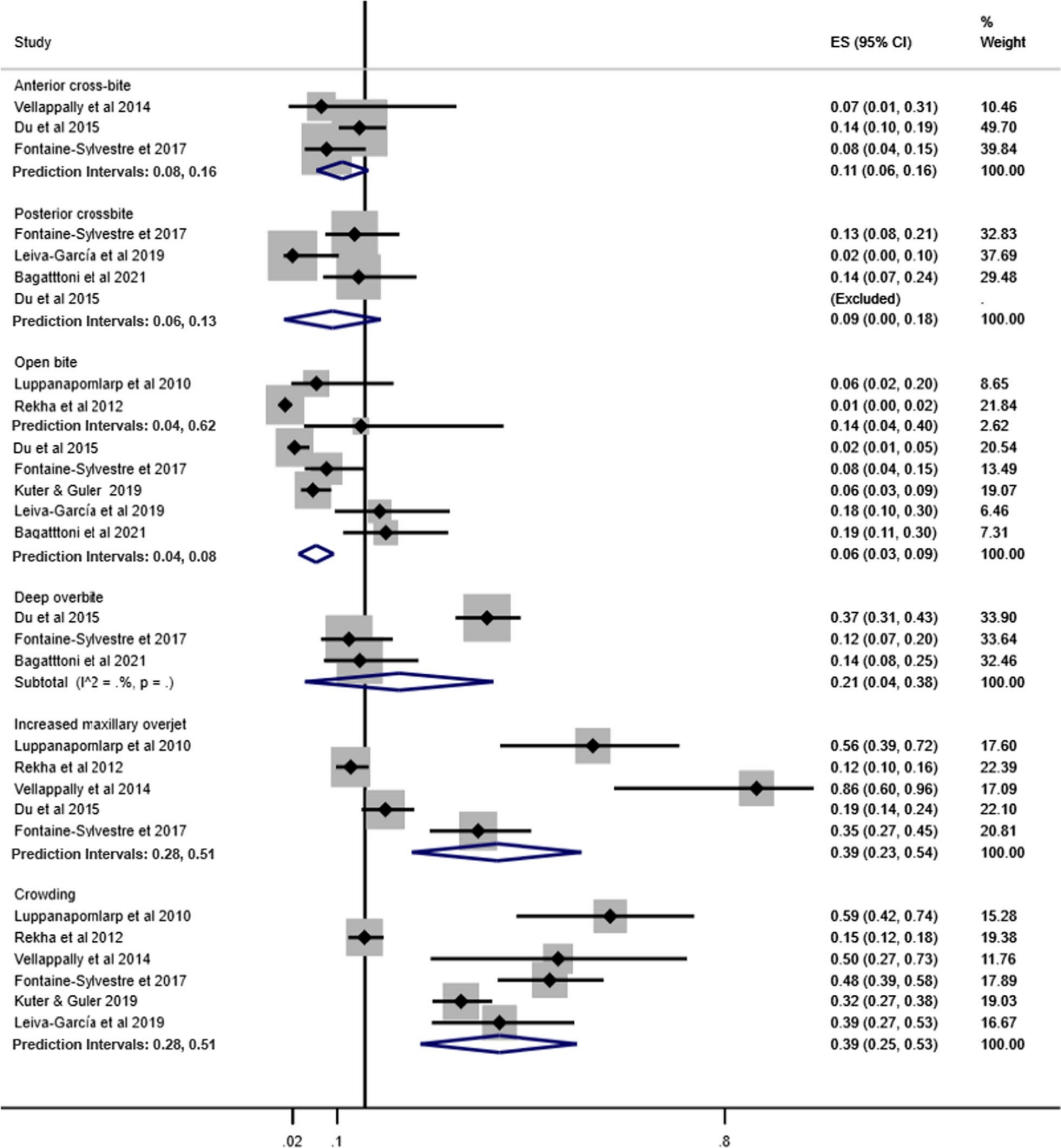
Prevalence of malocclusion characteristics in children and adolescents with ASD

**Fig. 8:**
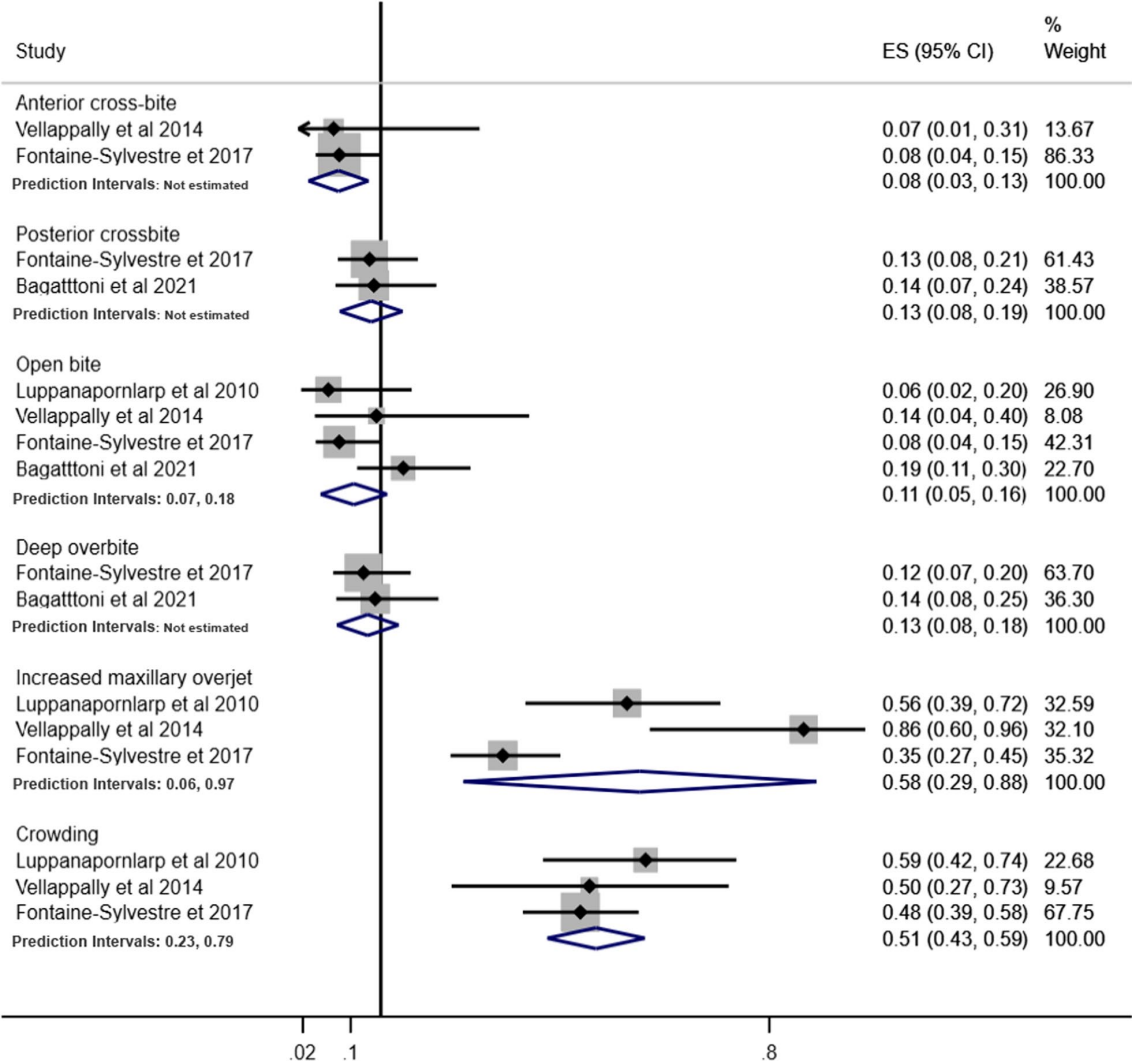
Prevalence of malocclusion characteristics in individuals with ASD without history of orthodontic treatment

**Fig. 9:**
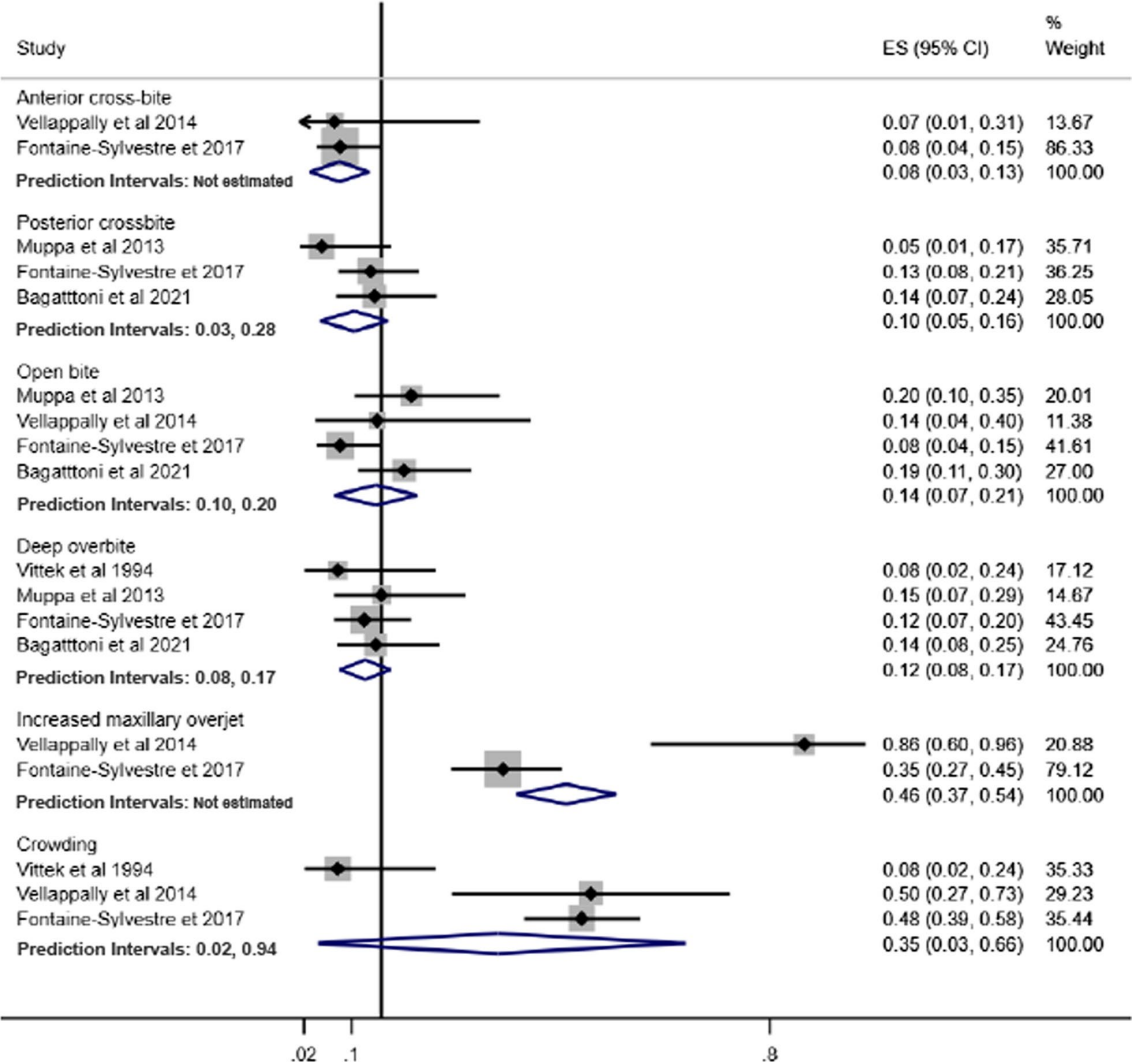
Prevalence of malocclusion characteristics in individuals with ASD involving studies that excluded or provided information about other syndromes and medical conditions

Pooled prevalence of malocclusion characteristics according to children and adolescents, history of orthodontic treatment and studies that excluded or provided information about other syndromes and medical conditions are presented in Figs. 7, 8 and 9, respectively.

The pooled prevalence of malocclusion characteristics including all studies tended to be lower than the subgroup analyses. For instance, the pooled prevalence of posterior cross-bite including all studies was 6%, while in the subgroup analyses, prevalence estimates were children and adolescents (9%), history of orthodontic treatment (13%) and studies that excluded or provided information about other syndromes and medical conditions (10%). Similarly, the pooled prevalence of increased maxillary overjet for all studies, and the subgroup analyses of studies that excluded previous orthodontic treatment and studies that excluded or provided information about other syndromes and medical conditions were 39%, 58% and 46%, respectively.

### Association between ASD and malocclusion

**Fig. 10:**
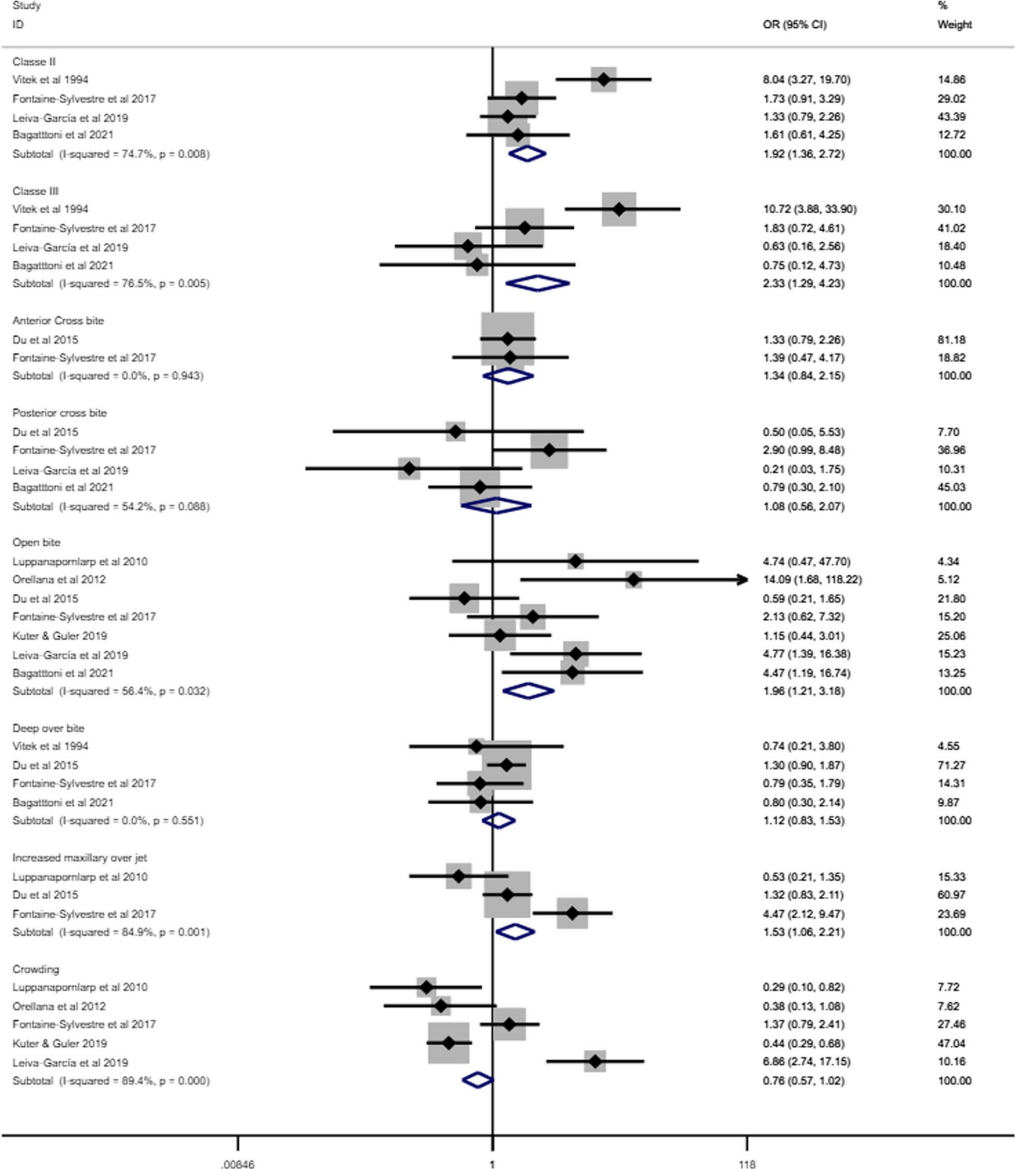
Forest-plot for association between ASD and malocclusion

**Fig. 11:**
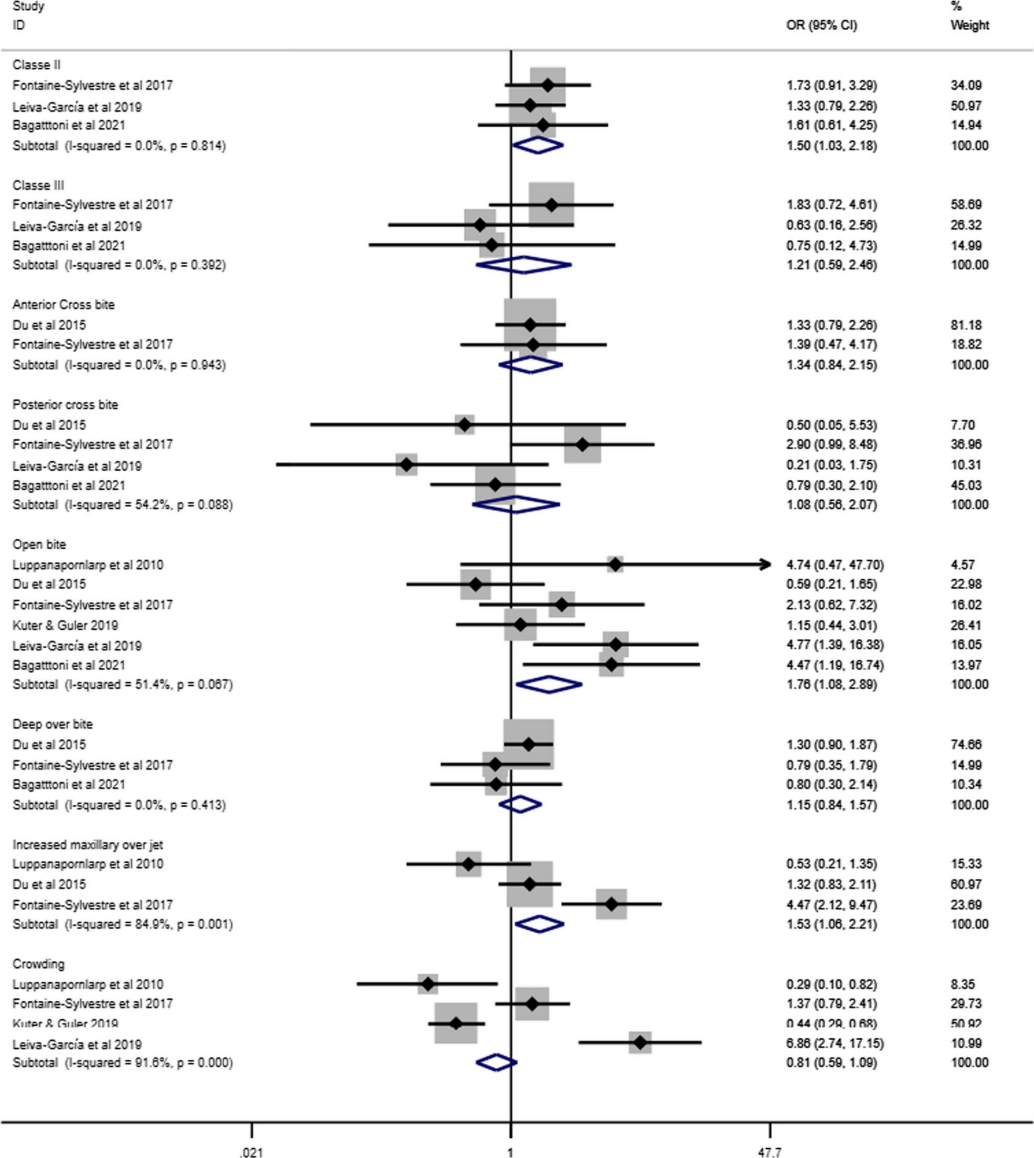
Association between ASD and malocclusion in children and adolescents with ASD

**Fig. 12:**
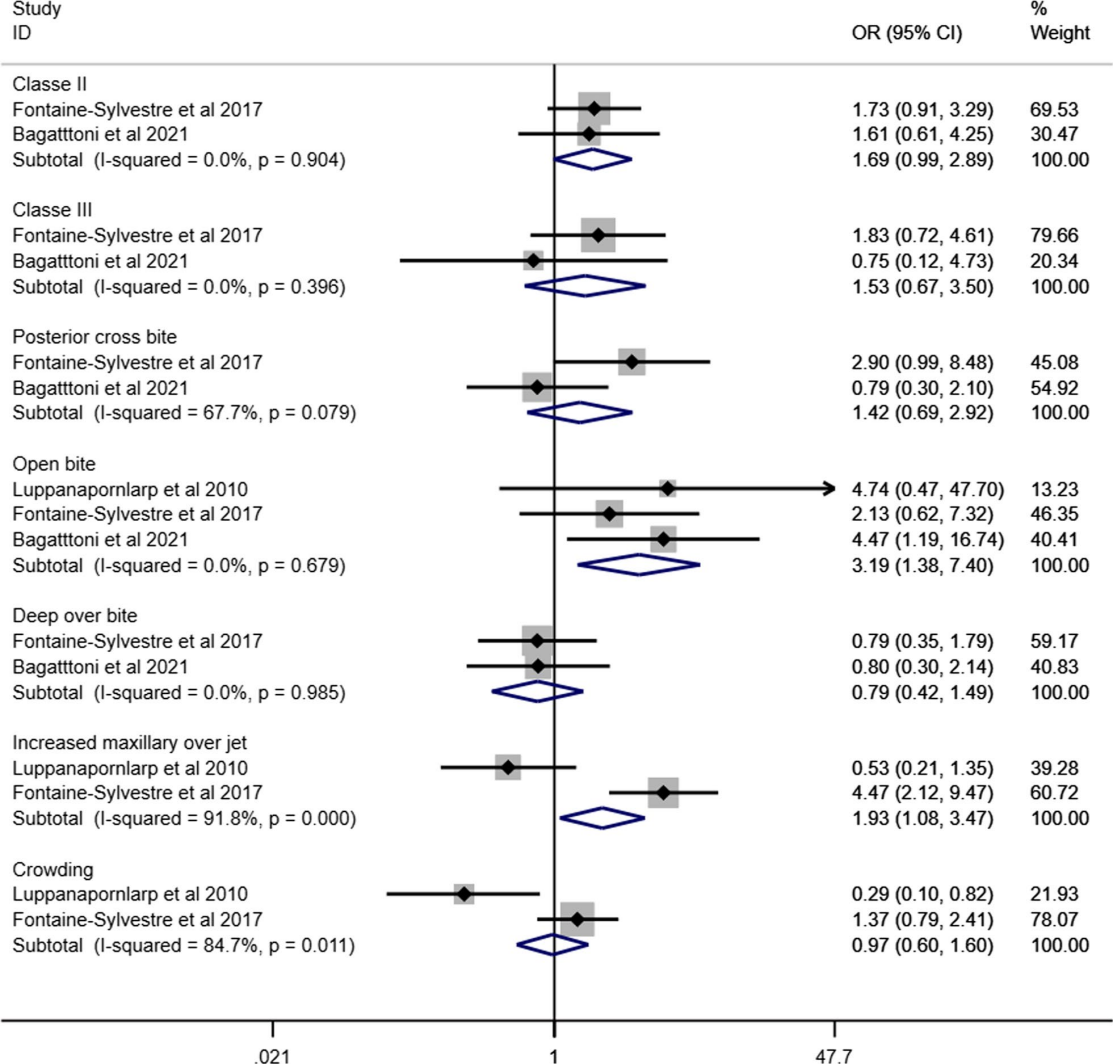
Association between ASD and malocclusion in individuals without history of orthodontic treatment

**Fig. 13:**
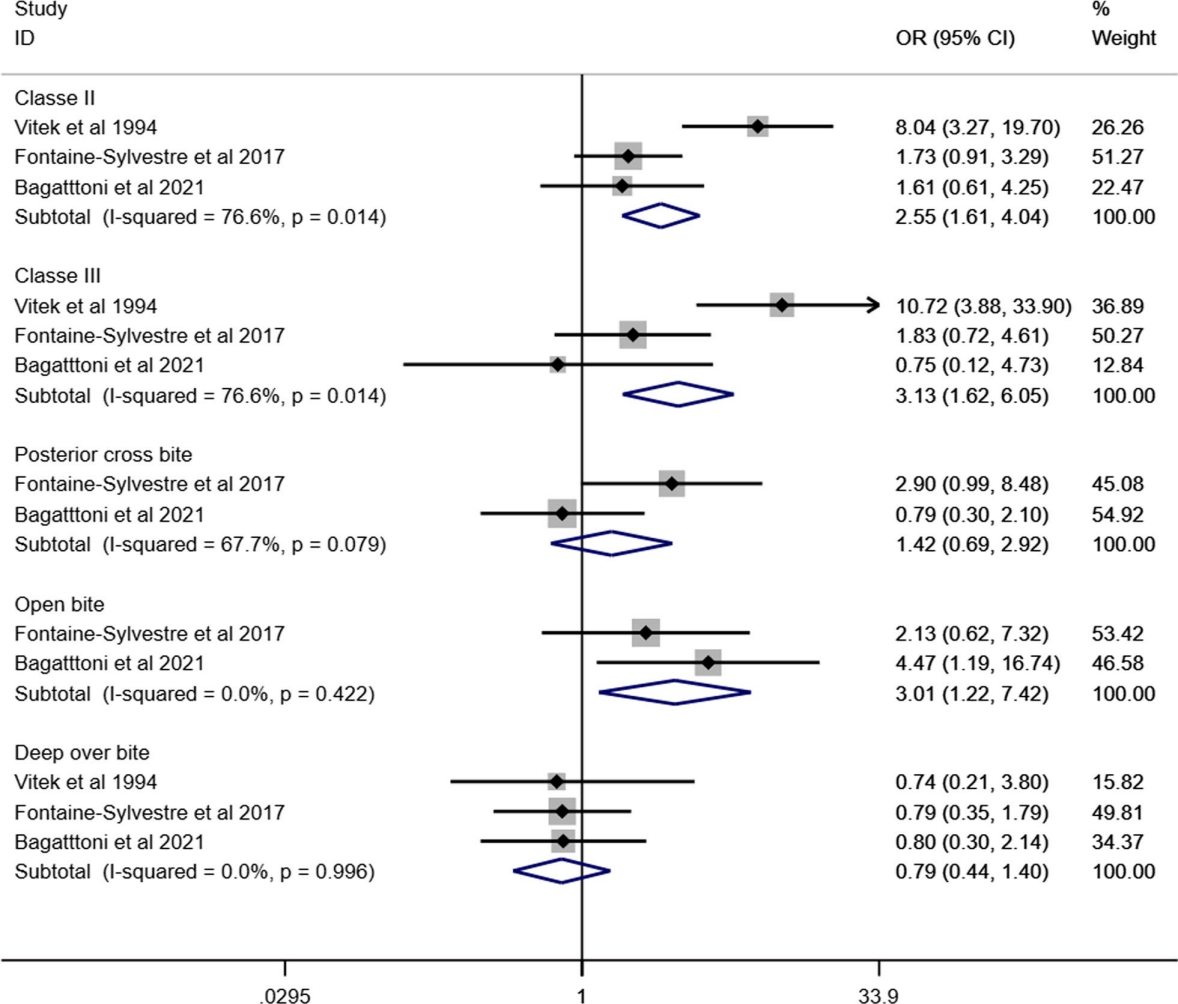
Association between ASD and malocclusion involving studies that excluded or provided information about other syndromes and medical conditions

Figure 10 presents the forest plot of the meta-analyses assessing the association between different malocclusion characteristics and ASD based on data extracted from eight articles involving 848 individuals with ASD and 9554 individuals without ASD [35, 38, 40, 44, 46, 48, 49, 52]. Individuals with ASD had significantly higher odds of Angle’s class II (OR 1.92, 95% CI 1.36–2.72), Angle’s Class III (OR 2.33, 95% CI 1.29–4.23) and open bite (OR 1.96, 95% CI 1.21–3.18) than those without ASD. The odds of having increased maxillary overjet were 53% higher for individuals with ASD than those without ASD (OR 1.53, 95% IC: 1.06–2.21). Heterogeneity was observed in Angle’s Class II (I^2^ = 75%), Angle’s Class III (I^2^ = 77%), open bite (I^2^ = 56%), increased maxillary overjet (I^2^ = 85%), and crowding (I^2^ = 89%). The association between ASD and malocclusion characteristics in subgroup analyses is presented according to children and adolescents (Fig. 11), history of orthodontic treatment (Fig. 12) and studies that excluded or provided information about other syndromes and medical conditions (Fig. 13). The association of ASD with Angle’s Class II and Angle’s Class III was not significant when pooling data from studies excluding participants with previous orthodontic treatment.

## Discussion

The present systematic review and meta-analysis was conducted to examine the following research questions: (i) what are the malocclusion characteristics and the most common occlusal disorders of individuals with ASD? and (ii) do individuals with ASD have a greater likelihood of malocclusion than those without ASD? It was hypothesised that individuals with ASD had more severe occlusal deviations than those without ASD. Overall, 18 primary studies addressing these research questions were identified. These studies used six different clinical occlusal measures and two malocclusion classification systems. According to the first research question, our findings demonstrated that occlusal deviation in individuals with ASD was represented by horizontal occlusal disorders and reduced spacing, including increased maxillary overjet and crowding. Vertical and transversal occlusal problems, represented by a posterior crossbite and open bite, were less commonly found in these individuals, although the likelihood of an open bite among individuals with ASD was significantly higher than among individuals without ASD. The occurrence of Angle’s Class II was more than three times higher than Angle’s Class III in individuals with ASD. In addition, 38% of individuals with ASD were classified as highly desirable and in need of treatment for malocclusion according to DAI [53]. The second research question and the study’s hypothesis were confirmed into some extent. Individuals with ASD had higher odds of Angle’s Class II, Angle’s Class III, open bite, and increased maxillary overjet than individuals without ASD. The remaining four malocclusion characteristics investigated were not associated with ASD.

There is a dearth of systematic reviews aiming to characterize the characteristics and prevalence of malocclusion in individuals with ASD as well as investigating the relationship between malocclusion and ASD. Most of the previous review papers on oral health status and ASD have assessed dental caries and periodontal disease. The only previous review on this topic indicated a prevalence of malocclusion in children with and without ASD of 60% and 40%, respectively. However, these figures did not differ statistically [9].

According to our findings, individuals with ASD are at higher risk of malocclusion. It could be argued that the influence of ASD on malocclusion might be explained by behavioural factors [26, 54]. For example, children diagnosed with ASD had lower breastfeeding rates, were weaned earlier, had a preference for liquid foods and transitioned later to solid foods [54]. The lack of adequate dietary masticatory stimulation during development directly influences human craniofacial growth and consequently may predispose the occurrence of occlusal deviations [55]. Moreover, individuals with ASD had higher rates of persistent parafunctional habits, including mouth breathing and biting objects than those without ASD [26]. Mouth breathing, for instance, is closely associated with an open bite. Mouth breathers may also exhibit vestibular inclination of the upper incisors and clockwise rotation of the mandible, contributing, in part, to the increased maxillary overjet [56, 57] and may also exhibit deformity of the dental arches, which may lead to tooth-size/arch-length discrepancy and space problems [58, 59]. Finally, the challenges involved in the management of individuals with ASD in the dental setting, may lead to the late diagnosis of malocclusions and preclude the early treatment of any occlusal alteration [60].

A relevant aspect of the present meta-analysis worth mentioning is the use of different malocclusion measures, which considered distinct transversal, horizontal and vertical occlusal deviations. Thus, data were combined according to type of malocclusion, enabling identification of the prevalence of different occlusal problems as well as malocclusions associated with ASD. The use of a random effects model in meta-analyses of observational studies is considered a valid strategy to account for some of the between-study variation. Heterogeneity was observed in some of the meta-analyses in terms of prevalence of malocclusion and on the association between ASD and malocclusion notwithstanding. This might be considered an expected finding since all studies included in this review were cross-sectional designs, with frequent methodological discrepancies.

The meta-analyses on the link between ASD and malocclusion characteristics only included eight studies. The paucity of primary studies assessing the influence of ASD on malocclusion characteristics may have affected the statistical power of the quantitative synthesis, particularly in the subgroup analyses of studies including only children and adolescents, studies excluding individuals with history of orthodontic treatment, and studies that excluded or provided information about other syndromes and medical conditions. The need for further studies is paramount to ascertain the role of ASD on malocclusion. However, the use of robust methodology in future research is essential to reach valid conclusions. One suggestion is that future studies evaluating the possible influence of ASD on malocclusion should use representative samples of individuals with ASD, select an adequate group for comparison, and assess potential confounding factors, including previous orthodontic treatment, other syndromes, parafunctional habits, and history of feeding habits.

The monitoring of preventive and risk factors for malocclusion as well as orthodontic treatment should be carried out by multidisciplinary health teams, including orthodontists, paediatric dentists, paediatricians, occupational therapists, speech and language therapists and psychologists [60]. Multidisciplinary approaches could also enhance oral health related quality of life along with the functional aspects of oral health. The difficulties and barriers to accessing specialized dental care, including orthodontic care, among individuals with ASD in most countries reinforces the importance of early diagnosis of malocclusion for children diagnosed with ASD. Moreover, the benefits of orthodontic treatment on masticatory and speech function, orofacial musculature as well as quality of life supports the development of orthodontic therapies for individuals with ASD [32].

There are methodological limitations of this systematic review. First, all included studies are cross-sectional which imposes important constraints because they do not infer cause and effect and are only a snapshot in time. Although this might not be considered a meaningful problem since ASD (the exposure) is an innate exposure and malocclusion can only be observed after the first years of life, most research on this topic adopted an exploratory approach. Therefore, testing the association between ASD and malocclusion was limited in most studies due to lack of appropriate comparison groups and an insufficient analytical approach.

Second, only five primary studies included in this systematic review and meta-analysis investigated whether individuals with and without ASD were already treated for occlusal deviations. The remaining studies did not inform whether or not individuals received orthodontic treatment, if the malocclusion was corrected among those who were treated for malocclusion. Eight studies recorded the occurrence of other syndromes and medical conditions, but failed to discuss any relationship. Moreover, age group was a selection criterion in only six studies. This may suggest the confounding effect of previous orthodontic treatment and other factors in studies reporting the association between ASD and malocclusion. No manuscripts conducted power calculations to estimate the sample size, leaving the studies subject to type I and type II errors. This means that the magnitude of any significant difference and precision and variance within the samples is unclear.

Third, there was no information and discussion around syndromes that may be associated with ASD, the level of commitment of the individual’s autistic spectrum, dietary patterns and tooth loss of individuals with ASD, as well as the facial profile and malocclusion of their parents and genetic influences. Most studies included in this review addressed ASD as a homogenous condition, failing to report the interplay of associated syndromes, the level of commitment of the individual’s autistic spectrum, behavioural mechanisms (eg. use of bottle feeding) and parental factors which may exert an effect on facial and skeletal morphology and increase the prevalence of malocclusion. A more nuanced approach, distinguishing between essential autism and complex (syndromic) autism, across different degrees of the ASD spectrum for individuals, could be a potential starting point for future research. Furthermore, behavioural factors and parental characteristics related to malocclusion should be collected in the forthcoming studies.

Fourth, according to the eligibility criteria for the present study there were no restrictions regarding participant age limits. This approach was adopted to identify and include all relevant publications on this topic. Although published studies involved mostly children and adolescents, at least five studies included adults. Conducting a sub-group analysis for adults was not possible due to the limited number of included studies.

Finally, 13 of the 18 studies included were classified as having critical risk or serious risk of bias due to the limitations, along with other methodological flaws. Additionally, primary studies did not consider the ASD spectrum when reporting malocclusion characteristics in individuals with ASD. Thus, future studies should acknowledge and overcome the methodological limitations highlighted in this systematic review and meta-analysis consider the wide spectrum of ASD.

Our study has demonstrated the prevalence of different malocclusion characteristics in individuals with ASD varied meaningfully according to different malocclusion measures. Angle’s Class II, DAI elective treatment need, DAI mandatory need treatment, increased maxillary overjet and crowding were the most common occlusal deviations. The present findings also provide evidence to support specific occlusal deviations, including Angle’s Class II, Angle’s Class III, open bite, and increased maxillary overjet were more prevalent among individuals with ASD than those without ASD. Early diagnosis of malocclusion may assist in prompt intervention and improvement of the oral health of people with ASD across the life course.

## Data Availability

The datasets used and/or analyzed during the current study are available from the corresponding author on reasonable request.
